# Practical Aspects of Esophageal Pressure Monitoring in Patients with Acute Respiratory Distress Syndrome

**DOI:** 10.3390/jpm13010136

**Published:** 2023-01-10

**Authors:** Pavel Dostal, Vlasta Dostalova

**Affiliations:** Department of Anesthesiology and Intensive Care Medicine, Charles University, Faculty of Medicine in Hradec Kralove, University Hospital Hradec Kralove, Sokolska 581, 500 05 Hradec Kralove, Czech Republic

**Keywords:** acute respiratory distress syndrome, mechanical ventilation, esophageal pressure, transpulmonary pressure, positive end-expiratory pressure

## Abstract

Esophageal pressure (P_es_) monitoring is a minimally invasive advanced respiratory monitoring method with the potential to guide ventilation support management. P_es_ monitoring enables the separation of lung and chest wall mechanics and estimation of transpulmonary pressure, which is recognized as an important risk factor for lung injury during both spontaneous breathing and mechanical ventilation. Appropriate balloon positioning, calibration, and measurement techniques are important to avoid inaccurate results. Both the approach of using absolute expiratory Pes values and the approach based on tidal Pes difference have shown promising results for ventilation adjustments, with the potential to decrease the risk of ventilator-induced lung injury.

## 1. Introduction

Esophageal pressure monitoring is a minimally invasive and clinically available method for estimating transpulmonary pressure [1], of which absolute values and changes are considered one of the main determinants of lung injury due to mechanical forces applied during mechanical ventilation [2]. The aim of this narrative review is to summarize the concept of esophageal pressure monitoring to assess transpulmonary pressures in patients with acute respiratory distress syndrome, its technical aspects of measurement, and its possible use for adjustment of the ventilator setting.

## 2. Transpulmonary Pressure, Pleural Pressure, and Esophageal Pressure

During mechanical ventilation, airway pressure distends the lung and chest wall in series. Increases in chest wall elastance, such as chest wall edema, kyphoscoliosis, and intraabdominal hypertension, are usually associated with increases in pleural pressure, as more force is necessary to distend the chest wall [3]; therefore, substantial and variable differences between airway and transpulmonary pressure may exist.

Transpulmonary pressure is a measure of lung stress, which is a distending force applied to lung structures. Changes in transpulmonary pressure are directly linked to changes in lung structure size—lung strain. If the stress and strain within the lung become unphysiological, the lung reacts by producing inflammatory cytokines and mediators, thereby initiating an inflammatory process within the lung parenchyma [4]. Although using a single value of transpulmonary pressure does not take into consideration lung inhomogeneity and ignores the role of respiratory rate and inspiratory flow in the development of ventilator-induced lung injury (VILI), transpulmonary pressure represents a physiologically sound safety limit for mechanical ventilation that should be measured and targeted, at least in patients with severe ARDS [2].

Transpulmonary pressure (P_L_) is defined as the pressure that corresponds to the pressure distending the lung, that is, the difference between airway pressure (P_aw_) and pleural pressure (P_pl_). If this pressure is measured under static conditions during a sufficiently long inspiratory or expiratory pause, it corresponds to the difference between alveolar pressure (P_alv_) and P_pl_.

Transpulmonary pressure changes during the respiratory cycle. Inspiratory and expiratory transpulmonary pressures are usually considered separately as safety limits or targets during mechanical ventilation [2,5]. Owing to gravitationally dependent pleural pressure differences (the so-called vertical pleural pressure gradient), there are also gravitationally dependent differences in transpulmonary pressures between different lung regions. During passive mechanical ventilation, lower pleural pressure values are expected in the non-dependent lung regions. Therefore, non-dependent lung regions are exposed to higher and dependent regions to lower inspiratory transpulmonary pressures. During spontaneous breathing, the respiratory muscles, mainly the diaphragm, generate negative pleural pressure, which is most negative close to the dorsal regions of the diaphragm [2]. Therefore, dependent lung regions are exposed to the highest inspiratory transpulmonary pressure during spontaneous breathing [6]. During pressure support ventilation, tidal volume distribution may be similar to spontaneous breathing; however, higher pressure support levels modify the distribution of ventilation and pleural pressure, which may be very close to the passive condition [7]. P_es_ is considered an estimate of the pleural pressure close to the balloon position in the chest. In humans, it approximates mid-thoracic pleural pressure [8]. Therefore, it underestimates pleural pressure in the most dependent lung regions and overestimates it in non-dependent lung regions. The vertical gradient of pleural pressure is significantly greater in injured versus normal lungs (1.8 times) [8] and could be more than 10 cm H_2_O in human cadavers in the supine position [6]. Consequently, P_L_ calculated as the difference between P_aw_ and P_es_ could be overestimated in the most dependent and underestimated in the most non-dependent lung regions by more than 5 cm H_2_O [8].

Direct subtraction of the absolute values of Paw and Pes (Table 1), the so-called directly measured P_L,_ is currently considered an accurate estimation of P_L_ in the mid-chest and dependent regions, with implications for the positive end-expiratory pressure (PEEP) setting [8] to decrease the risk of derecruitment in the dependent lung. As previously described, this method underestimates P_L_ in non-dependent lung regions.

The release-derived transpulmonary pressure method (Table 1) is the reference method for calculating total end-inspiratory transpulmonary pressure, a surrogate of lung stress. Its calculation requires measuring P_es_ at a P_aw_ equal to the atmospheric pressure, usually during disconnection from the ventilator. Although the P_L_ calculated using the release-derived method probably reflects total lung stress better than other methods used to estimate P_L_ [9], disconnection from the ventilator could be associated with sudden deterioration of respiratory function. Therefore, the release-derived calculation of transpulmonary pressure remains mainly a research tool.

The elastance-derived transpulmonary pressure method (Table 1) estimates the total end-inspiratory P_L_, multiplying the airway plateau pressure (P_plat_) by the ratio between the lung and the total respiratory system elastance [9,10]. This method of P_L_ calculation is currently considered the method of choice for the clinical estimation of inspiratory P_L_ in non-dependent regions [8], and it is considered an indirect estimate of total lung strain and the risk of overdistension. A possible limitation of this method is the nonlinear behavior of lung and chest wall elastance at the extremes of the pressure/volume curve of the respiratory system [9].

The P_es_ value can also be used to calculate so-called lung or transpulmonary driving pressure, which is considered another important indicator of possible lung injury during both spontaneous and mechanical ventilation. Transpulmonary driving pressure is calculated either as the difference between end-inspiratory and end-expiratory transpulmonary pressures, or as the difference between airway driving pressure and the so-called chest wall driving pressure, which is the difference between the measured inspiratory and expiratory esophageal pressures (Table 1) [11]. Transpulmonary driving pressure is considered an indirect estimate of dynamic lung strain.

**Table 1:** Suggested methods to calculate inspiratory transpulmonary pressure, expiratory transpulmonary pressure, total transpulmonary pressure, and transpulmonary driving pressure. All pressures must be measured under static conditions after the airway occlusion maneuver. Adapted from Mieto et al. [9] and Grieco et al. [11].

Parameter	Method	Computation
End-inspiratory P_L,_	Elastance-derived	P_L_ = P_plat_ x E_L_/E_rs_ = P_plat_ x [(P_plat_ − P_esplat_) − (P_awPEEP_ − P_esPEEP_)]/(P_plat_ − P_awPEEP_)
End-inspiratory P_L_ (reference method)	Release-derived	P_L_ = (P_plat_ − P_awATM_) − (P_esplat_ − P_esATM_)
End-expiratory P_L_	Direct method	P_L_ = P_awPEEP_ − P_esPEEP_
Driving P_L_	Direct method	DP_L_ = (P_plat_ − P_esplat_) − (P_awPEEP_ − P_esPEEP_), or DP_L_ = (P_plat_ − P_awPEEP_) − (P_esplat_ − P_esPEEP_)

P_L_—transpulmonary pressure; P_plat_—plateau pressure; PEEP—positive end-expiratory pressure; E_L_—lung elastance; E_rs_—respiratory system elastance; P_esPEEP_—esophageal pressure measured at PEEP; P_esplat_—esophageal pressure measured at plateau pressure; P_esATM_—esophageal pressure at atmospheric pressure; P_awPEEP_—airway pressure at PEEP.

## 3. Technical Aspects of Esophageal Pressure Measurement

P_es_ measured using the balloon technique is considered the best noninvasive surrogate of pleural pressure available at the bedside in critically ill patients. As it is an indirect estimate of pleural pressure, possibly affected by some artifacts, the correct technique of catheter insertion, balloon positioning, filling, and validation testing are important for obtaining reliable results [12].

### 3.1. Ballon Catheter Insertion and Positioning

Some esophageal balloons are integrated into a traditional nasogastric feeding tube, whereas others are carried by a thin, dedicated tube [12]. The insertion of the catheter is similar to that of a nasogastric tube. The integrity of the catheter and balloon should be checked by inflation of the catheter using the specific recommended amount of air through the balloon port. Then, the catheter and its deflated balloon are lubricated and inserted through the nostril into the esophagus and stomach. Depth markers on the catheter aid orientation and assessment of the insertion depth. The intragastric position of the balloon may be confirmed after the balloon port is connected to the pressure monitoring device and inflation of the balloon with the recommended volume. The position of the balloon in the stomach can be confirmed by visualization of the positive deflections of the balloon pressure during gentle external compressions of the left upper abdominal quadrant [12]. Although the presence of a nasogastric tube does not affect the reliability of the measurement [13,14], insertion of a thin balloon catheter could be more difficult. If the nasogastric tube is already in place, it may be necessary to remove it before inserting the balloon catheter.

After insertion of the balloon catheter into the stomach and inflation with the volume of air recommended by the manufacturer, the catheter is slowly withdrawn into the mid-distal esophagus. Catheters usually have depth markers to aid in positioning the balloon, and the depth at which the balloon should be placed can be estimated on the basis of the distance from the nostril to the ear tragus to the xyphoid. In almost all cases, the correct distance between the nostril and distal end of the balloon ranges from 35 to 45 cm [12]. Changes in the P_es_ waveform are observed during the slow and stepwise withdrawal of the balloon catheter. A sudden change in baseline pressure is expected when the balloon transitions from the abdomen to the chest [12]. Positive deflections of P_es_ should be visible in patients on positive pressure ventilation, and negative deflections in spontaneously breathing patients. Typical cardiac artifacts usually appear in the P_es_ waveform, and their presence suggests the balloon’s position beneath the heart. If cardiac oscillations are excessive and prevent reliable assessment of the tidal swing of the P_es_, the catheter can be carefully pulled further back, but the position of the balloon in the upper esophagus should be avoided [12]. An additional check on the correct positioning can be performed by examining the waveforms. The chest wall has linear elastic behavior; thus, a linear relationship between the volume and pleural pressure is expected. Therefore, if the esophageal balloon is surrounded by pleural pressure (i.e., it is in the correct position), the esophageal balloon pressure and volume curves should have a similar shape (not amplitude) on the monitoring device or ventilator screen [12].

### 3.2. Ballon Filling and Validation Testing

Balloon catheters produced by different producers differ in terms of the material used, volume, length, and elastance. Therefore, the use of balloon-specific filling volumes is required because underfilled balloons tend to underestimate and overfilled balloons tend to overestimate the real P_es_ value [13,15]. Larger balloons usually have a wider range of adequate filling volumes, at which point the balloon wall elastance does not affect the measured P_es_ values [13]. It enables adjustment of the filling volume for other important factors, such as esophageal elastance (E_es_) and balloon-surrounding pressure. Higher surrounding pressures require higher filling volumes to reliably measure tidal P_e_s swings [13].

**Figure 1:**
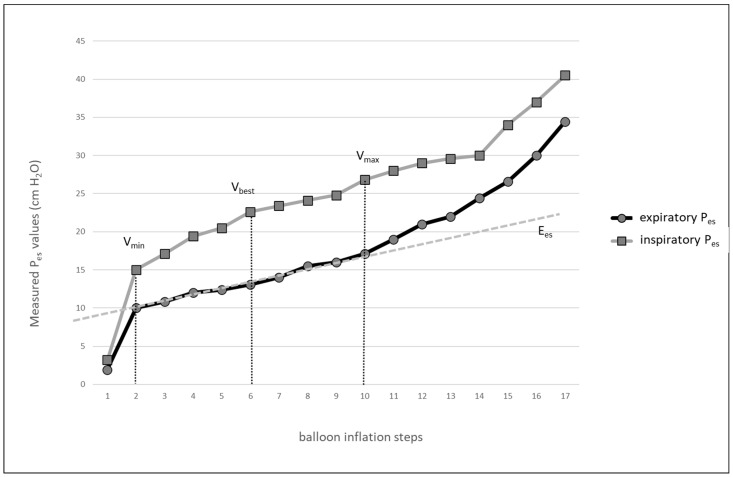
An example of the balloon pressure-volume curve obtained in an obese patient with influenza A pneumonia and congestive heart failure. Inflation steps are shown on x axis. E_es_—esophageal elastance; V_min_—volume of the balloon at the beginning of the linear part of balloon pressure-volume curve; V_max_—volume of the balloon at the end of the linear part of balloon pressure-volume curve; V_best_—lowest volume of the balloon with largest tidal difference of esophageal pressure; P_es_—esophageal pressure.

To overcome the problem of the sub- or supra-optimal filling volume of the balloon, inter-individual differences in esophageal elastance, and the effects of the surrounding pressure, a technique of in vivo calibration of Pes has recently been proposed [15]. Briefly, the balloon is inflated stepwise using small aliquots of air (Figure 1). P_esplat_ and P_esPEEP_ are recorded at each step. Further balloon inflation is interrupted if a sudden substantial increase in the P_es_ baseline is observed. The smallest inflation volume with the largest tidal swing of P_es_ below the balloon volume, associated with a sudden baseline change in P_es_, is considered the best volume (V_best_).

**Figure 2:**
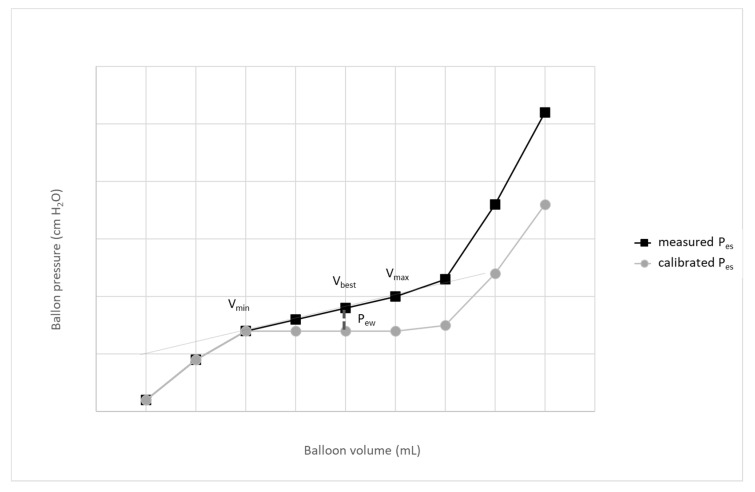
Effect of correction of esophageal elastance. A simplified scheme; expiratory P_es_ values are shown only. P_ew_—pressure generated by the distension of esophageal wall; V_min_–volume of the balloon at the beginning of the linear part of the balloon pressure-volume curve; V_max_—volume of the balloon at the beginning of the linear part of the balloon pressure-volume curve.

To compensate for the baseline changes of P_es_ at higher inflation volumes, it was suggested to subtract from the measured value of P_es_ the pressure generated by the distension of the esophageal wall—esophageal recoil pressure (P_ew_). This pressure can be calculated from the elastance of the linear part of the pressure-volume curve of the balloon: P_ew_ = (V_best_ − V_min_) × E_es_, where V_best_ in mL is the balloon volume with the largest tidal swing of P_es_,V_min_ in mL is the minimum filling volume of the balloon at the beginning of the linear part of the balloon pressure-volume curve (Figure 2), and E_es_ in cm H_2_O/mL is calculated using the least square fitting method from Pes values [15,16]. E_es_ can also be estimated using a simplified method based on the P_es_ values obtained at V_min_ and V_max_ [17]. Using this approach, E_es_ is calculated as the difference in P_es_ at V_max_ and V_min_ divided by the difference between V_max_ and V_min_, and calibrated P_es_ is calculated as the difference between the measured P_es_ at V_best_ and P_ew_ at V_best_ (Table 2, Figure 2). All calculations can be done using an appropriate table calculator.

To check the position of the balloon and its appropriate filling, a validation test, also called the occlusion test or Baydur test, should be performed during airway occlusion [12]. During the end-expiratory occlusion maneuver, the occluded airway impedes changes in the intrathoracic volume. Patient breathing effort, or gentle sternal compression, leads to changes in the intrathoracic pressure that are fully and equally transferred into the airways, pleural space, and esophagus. In the case of a properly placed and filled balloon catheter, the changes in P_es_ (ΔP_es_) are expected to be equal to the changes in P_aw_ (ΔP_aw_). The target value of the ΔP_es_/ΔP_aw_ ratio ranges from 0.9 to 1.1. If the ratio is outside this range, the balloon filling (first) and balloon positioning (second) should be rechecked [12].

There is ongoing discussion on whether balloon calibration is necessary for routine practice. Some authorities in the field of P_es_ monitoring state that using a balloon with a consistent working range of inflation volume helps to obtain consistent and accurate measurements. While the optimal inflation volume can be confirmed based on the pressure-volume characteristics of the balloon itself, calibration is time-consuming and not required in practice when using a balloon with a known working range. A simple observation of the P_es_ waveform should be sufficient to detect overinflation resulting in inaccurately high measured pressures secondary to the compliance of the balloon, whereas underinflation is associated with dampening of waveform variation [18]. The disadvantage of this simplified approach is that it does not correct for individual differences in esophageal elastance. Mojoli et al. [15] showed that the pressure generated by the esophageal wall could range from 0 to +6 cm H_2_O and that the optimal filling volume range was highly variable among different patients and conditions, ranging from 0.5 to 6 mL in their series. The ventilator setting and body mass index (BMI) modify the optimal filling volume [15,19]. This suggests that the esophageal catheter filling volume should be adapted to the intrathoracic pressure condition of any patient [15]. Using the simplified calibration method suggested by Sun et al. [17], the measured P_es_ value at V_best_ is corrected to obtain the P_es_ value at V_min_. Therefore, if the value of the end-expiratory transpulmonary pressure is of interest, visual identification of V_min_ from the balloon pressure-volume curve and using a corresponding P_es_ value is probably sufficient for clinical use. For a more reliable estimation of transpulmonary driving pressure and end-inspiratory transpulmonary pressure, identification of V_best_ and the corresponding end-inspiratory and end-expiratory P_es_ values is necessary. The calibration procedure is not complicated and is clinically feasible [19].

In summary, stepwise inflation of the balloon with an appropriate amount of air (0.2–1.0 mL) according to the type of balloon catheter can be recommended. If the expiratory P_es_ value after the inflation step remains close to a previous value (difference within 1.0 cm H_2_O), the previous volume of the balloon is used to measure the expiratory P_es_ value, and no correction of the measured value is necessary. Adjustment of the balloon inflation is possible based on the results of a validation test during airway occlusion. V_best_ estimation is necessary for a more accurate estimation of the inspiratory transpulmonary pressure in nondependent lung regions.

### 3.3. Artifacts, Errors, and Rules during the Pes Measurement

Esophageal pressure is affected by many factors, including the patient’s weight, body position, muscle activity, heart weight, lung weight, ventilator setting, and presence of fluid in the pleural cavity [3]. Any change in these factors, such as setting higher PEEP values, influences P_es_. The effect of these factors cannot be considered artifacts or errors because they usually reflect either changed pleural pressure or a relative change in the esophageal position on a vertical thoracic axis.

The most important errors occur either because of improper measurement techniques or the effects of local factors, such as previously described cardiac oscillations or esophageal peristalsis, which could be detected as slow changes in the P_es_ baseline. The importance of optimal balloon positioning and calibration has been previously discussed. Possible overinflation due to previous insufficient balloon deflation may occur; therefore, complete and active deflation of the balloon with the syringe with the subsequent opening of the balloon port using a three-way stopcock to the atmosphere to allow equilibration of the atmospheric pressure and balloon pressure should be employed before balloon inflation. The mechanical properties of the balloon may change over time, and air leakage from the balloon or insufficient tightness of the connectors may occur. Incorrect calibration of the pressure transducer, particularly if an air-filled blood pressure transducer is used instead of a designated device, is also a potential source of error.

P_es_ should be measured at the body position where the patient is ventilated. Position changes are associated with changes in the P_es_ value, with high interpersonal variability [3]. The prone position is associated with lower P_es_ values in comparison with the supine position, possibly because of the diminished effect of mediastinum/heart weight, the more non-dependent position of the esophagus in the supine position, increased chest wall elastance, and a lower vertical pleural pressure gradient in the supine position. Changes in the setting of the ventilator, mainly the selection of a different PEEP level, require repeating the measurement of P_es_ [15,19,20].

## 4. Ventilatory Setting Based on Transpulmonary Pressure in Passive ARDS Patients

Esophageal pressure monitoring allows for the differentiation of chest wall, lung, and respiratory system mechanics, and there is ongoing interest in using P_es_ values for PEEP titration, monitoring of parenchymal lung stress, limiting peak end-inspiratory transpulmonary pressures, and monitoring ventilator synchrony [1,18].

### 4.1. PEEP Setting Targeting Expiratory Transpulmonary Pressure

Elevation of pleural pressure occurs in dependent regions due to increased chest wall weight in patients with obesity, chest wall edema, or pleural effusions. Increased lung weight or elevated intra-abdominal pressure may lead to lung derecruitment, mainly in dependent lung regions, increased lung elastance, and hypoxemia. It was hypothesized that, if the pleural pressure is greater than the airway/alveolar pressure, the application of PEEP may be helpful [18] in preventing lung derecruitment. The EPVent [5] and EPvent2 [21] studies investigated the use of esophageal manometry to titrate PEEP. Although the results of the EPvent study suggested a possible benefit of a strategy using a PEEP setting based on expiratory transpulmonary pressures, the EPvent2 study did not show a clear benefit compared to a high-PEEP strategy. Further analysis [22] of EPvent2 data suggested a benefit when end-expiratory P_L_ was maintained in a tight physiological range of − 2 to + 2 cm H_2_O with PEEP adjustment. Based on the results of this mechanistic analysis, proponents of this approach suggest titrating PEEP to obtain expiratory P_L_ close to zero [18,21]. The benefits of this approach should be expected in obese patients and patients with intra-abdominal hypertension. Although this approach is appealing, it has not been tested in a prospective randomized clinical trial; performed trials targeted an end-expiratory P_L_ between 0 cm H_2_O and +6 cm H_2_O, depending on the inspiratory fraction of the oxygen (FiO_2_) requirement. Strong evidence from experimental and clinical trials on mechanical ventilation suggests that the use of higher levels of PEEP could be beneficial only if the increment in PEEP is associated with improved compliance of the respiratory system [23], reasonable CO_2_ elimination, and stable patient hemodynamics [24,25]. This information should always be considered when attempting to adjust PEEP levels according to expiratory P_L_. Until further information from clinical trials is available, the evaluation of expiratory transpulmonary pressure could be useful as an indicator of the safety of the ventilator setting.

### 4.2. PEEP Setting Targeting Inspiratory Transpulmonary Pressure

This approach to setting the PEEP was used in patients considered ECMO candidates owing to severe H1N1 influenza pneumonia [26]. If the inspiratory P_L_ calculated using the release-derived method was below 25 cm H_2_O, using a constant tidal volume, PEEP was elevated to reach a so-called maximum physiological inspiratory P_L_ of 25 cm H_2_O. The inspiratory P_L_ value may be used either to limit the maximum value of PEEP or to personalize the targeting of the tidal volume. Recently, a safety limit for the maximum inspiratory transpulmonary pressure below 20–25 cm H_2_O was suggested [1,11], and a recent consensus statement suggested using inspiratory P_L_ as an indicator of the safety of ventilator settings [1].

### 4.3. Transpulmonary Driving Pressure and Safety

Respiratory system driving pressure is an important indicator of mechanical ventilation safety [23,27,28]. The upper limits for tidal changes in the transpulmonary pressure for patients with ARDS 10 to 12 cm H_2_O have recently been recommended for personalized targeting of the tidal volume [1,28]. The suggested limits for transpulmonary pressure are presented in Table 3.

## 5. The Other Possible Use for Measuring Esophageal Pressure in ARDS Patients

Measuring transpulmonary pressure is only one of the possible clinical applications of P_es_ monitoring; it can also be used to assess respiratory efforts in spontaneously breathing patients, evaluate patient-ventilator asynchronies, and compute transmural vascular pressures [1]. A detailed description of these topics is beyond the scope of the present review.

Although there are other clinically available alternatives, P_es_ monitoring could be used to identify and manage patients with a substantial risk of so-called patient self-inflicted lung injury (P-SILI) [29,30]. Recently, a novel noninvasive method for detecting excessively high respiratory effort using expiratory airway occlusion was developed [31]. In patients with an estimated high dynamic transpulmonary pressure (>15 cm H_2_O), the authors recommend considering the use of P_es_ monitoring to adjust patient management. P_es_ recording allows the detection of inappropriate inspiratory efforts. An absolute value of ∆P_es_ lower than 2–3 cm H_2_O is usually considered a sign of over assistance. Conversely, a ∆P_es_ higher than 8–12 cm H2O is considered a marker of under assistance [29,30].

## 6. Conclusions

Esophageal pressure monitoring is a minimally invasive and clinically appealing tool for personalized mechanical ventilation in ARDS patients. Both the approach of using absolute expiratory Pes values and that in which a tidal Pes difference is considered have shown promising results for ventilation adjustments, with the potential to decrease the risk of ventilator-induced lung injury.

## Figures and Tables

**Table 2:** Suggested steps to obtain a calibrated value of Pes. Adapted from Mojoli et al. [15] and Sun et al. [17].

Steps	Method
1st step	Perform a stepwise inflation of the balloon (0.2–1.0 mL steps, according to the balloon size), record P_es_ at different volumes of balloon.
2nd step	Using an appropriate table calculator, create the balloon pressure-volume curve to identify a linear part of the curve with V_min_ and V_max_, for further measurement select V_best_ with largest tidal change of P_es._
3rd step	Calculate the esophageal wall elastance: E_es_ = (P_esPEEPVmax_ − P_esPEEPVmin_)/(V_max_ − V_min_)
4th step	Calculate the esophageal recoil pressure: P_ewVbest_ = (V_best_ -V_min_) x E_es_
5th step	Calculate the calibrated esophageal pressure: calP_es_ = P_esVbest_ − P_ewVbest_

P_es_—esophageal pressure; V_best_—balloon inflation volume with highest tidal change of P_es_; V_min_—minimal balloon inflation volume on the linear part of esophageal pressure PV curve; V_max_—maximal balloon inflation volume on the linear part of esophageal pressure PV; E_es_—esophageal wall elastance; P_esPEEPVmax_—expiratory value of Pes measured at V_max_; P_esPEEPVmin_—expiratory value of esophageal pressure measured at V_min_; P_esVbes_t—esophageal pressure measured at V_best_; P_ewVbest_—esophageal recoil pressure at V_best_; calPes—calibrated value of esophageal pressure.

**Table 3:** Suggested limits of transpulmonary pressures in patients with ARDS. Adapted from Mauri at al. [1], Bayerdorf Kassis et al. [15], and Pelosi et al. [28].

Parameter	Limit
End-expiratory P_L_	±2 cm H_2_O
End-inspiratory P_L_	<20 cm H_2_O
Driving P_L_	<10–12 cm H_2_O
End-inspiratory P_L_ during recruitment maneuvers	≤25 cm H_2_O

P_L_—transpulmonary pressure.

## Data Availability

Not applicable.
